# *Bacillus pumilus* Reveals a Remarkably High Resistance to Hydrogen Peroxide Provoked Oxidative Stress

**DOI:** 10.1371/journal.pone.0085625

**Published:** 2014-01-20

**Authors:** Stefan Handtke, Rebecca Schroeter, Britta Jürgen, Karen Methling, Rabea Schlüter, Dirk Albrecht, Sacha A. F. T. van Hijum, Johannes Bongaerts, Karl-Heinz Maurer, Michael Lalk, Thomas Schweder, Michael Hecker, Birgit Voigt

**Affiliations:** 1 Institute for Microbiology, University of Greifswald, Greifswald, Germany; 2 Pharmaceutical Biotechnology, Institute of Pharmacy, University of Greifswald, Greifswald, Germany; 3 Institute of Biochemistry, University of Greifswald, Greifswald, Germany; 4 Centre for Molecular and Biomolecular Informatics (CMBI), Nijmegen Centre for Molecular Life Sciences, Radboud University Medical Center, Nijmegen, The Netherlands; and Division Processing and Safety, NIZO Food Research B.V., Ede, The Netherlands; 5 Department of Chemistry and Biotechnology, Aachen University of Applied Sciences, Jülich, Germany; 6 AB Enzymes GmbH, Darmstadt, Germany; 7 Institute of Marine Biotechnology, Greifswald, Germany; Loyola University Medical Center, United States of America

## Abstract

*Bacillus pumilus* is characterized by a higher oxidative stress resistance than other comparable industrially relevant Bacilli such as *B. subtilis* or *B. licheniformis*. In this study the response of *B. pumilus* to oxidative stress was investigated during a treatment with high concentrations of hydrogen peroxide at the proteome, transcriptome and metabolome level. Genes/proteins belonging to regulons, which are known to have important functions in the oxidative stress response of other organisms, were found to be upregulated, such as the Fur, Spx, SOS or CtsR regulon. Strikingly, parts of the fundamental PerR regulon responding to peroxide stress in *B. subtilis* are not encoded in the *B. pumilus* genome. Thus, *B. pumilus* misses the catalase KatA, the DNA-protection protein MrgA or the alkyl hydroperoxide reductase AhpCF. Data of this study suggests that the catalase KatX2 takes over the function of the missing KatA in the oxidative stress response of *B. pumilus*. The genome-wide expression analysis revealed an induction of bacillithiol (Cys-GlcN-malate, BSH) relevant genes. An analysis of the intracellular metabolites detected high intracellular levels of this protective metabolite, which indicates the importance of bacillithiol in the peroxide stress resistance of *B. pumilus*.

## Introduction

*Bacillus pumilus* is a Gram-positive, rod-shaped and endospore-forming bacterium closely related to the industrially relevant bacteria *Bacillus subtilis* and *Bacillus licheniformis*. *B. pumilus* represents a potential alternative host for the industrial production of enzymes. For the evaluation and optimization of fermentation processes with this organism a comprehensive knowledge on its physiology and stress adaptation is required.

During fermentation processes a variety of stresses (e.g. salt, heat and oxidative stress) can impair the fitness of the production host and the quality of the fermentation product [1]–[3]. *B. pumilus* strains are highly resistant against UV radiation and hydrogen peroxide, which may explain the finding of viable spores of *B. pumilus* in hostile environments such as the interior of the Sonoran desert basalt and spacecrafts [4], [5]. This natural potential and resistances of *B. pumilus* could be a major benefit for the improvement of industrial production strains, since oxidative stress can occur in all phases of fermentation processes [1]–[3].

Reactive oxygen species (ROS) such as superoxide (O_2_ **^·^** ^−^), hydrogen peroxide (H_2_O_2_) and hydroxyl radical (OH**·**) are successive one-electron-reduction products of molecular oxygen and therefore occur in all aerobically living organisms [3], [6], [7]. Increased ROS production that exceeds the cell defense capacity leads to oxidative stress in the cell and to the oxidation of nucleic acids, proteins and lipids [2], [3], [8]–[10].

In *B. subtilis*, the cellular defense against oxidative stress is ensured by the detoxification of harmful agents, protection of macromolecules and the repair or removal of damaged molecules. The oxidative stress response of this organism is regulated by specific transcriptional regulators, such as PerR, SigB, LexA/RecA, Spx and OhrR, as previously described in detail [11]–[13]. The oxidative stress response of *B. pumilus* differs significantly from the response in *B. subtilis*, as major oxidative stress genes of *B. subtilis* are missing in the genome of *B. pumilus*, such as the catalase KatA or alkyl hydroperoxide reductase AhpCF. For some of these genes no homologs could be found in the *B. pumilus* genome. This leads to the questions, which genes compensate the missing genes and are thus responsible for the oxidative stress resistance of *B. pumilus*. In this study we used a combination of proteomics, transcriptomics and metabolomics to investigate the individual peroxide stress response of *B. pumilus*.

## Materials and Methods

### 2.1 Strain, Media, Growth and Cell Sampling

*Bacillus pumilus* Jo2 (DSM 14395) was used for all experiments described in this study. Cells were grown aerobically at 37°C and 180 rpm in minimal medium containing 15 mM (NH_4_)_2_SO_4_, 8 mM MgSO_4_×7 H_2_O, 27 mM KCl, 7 mM Na-citrate×2 H_2_O, 50 mM Tris-HCl (pH 7.5) supplemented with 1.8 mM KH_2_PO_4_, 2 mM CaCl_2_, 1 µM FeSO_4_×7 H_2_O, 10 µM MnSO_4_×4 H_2_O, 4.5 mM glutamate, 0.2% w/v glucose and 0.04 µM biotin. Exponentially growing cells at an OD_500 nm_ of 0.6 were exposed to a final concentration of 2 mM hydrogen peroxide. Proteome samples were taken from unstressed cultures before and 10 as well as 30 minutes after exposure to hydrogen peroxide. Samples were pulse-labeled with L-[^35^S]-methionine for 5 min, as described by Hoi *et al.* [14]. Samples for preparative gels were prepared from unlabeled cells 30 and 60 min after exposure to H_2_O_2_ [14]. Preparative gels were used only for spot identification via mass spectrometry.

Samples for RNA extraction were taken before (control) and 3 and 8 min after addition of H_2_O_2_. Cell samples for RNA extraction were mixed with 0.5 volumes of ice-cold killing buffer (20 mM Tris-HCl pH 7.5, 5 mM MgCl, 20 mM NaN_3_), and immediately harvested at 10000×g for 5 min at 4°C.

### 2.2. Scanning Electron Microscopy

For the scanning electron microscopy, the cells were separated from the culture medium by filtration through a 0.2 µm pore size polycarbonate filter. The filter were placed in fixation solution (1% glutaraldehyde, 4% paraformaldehyde, 50 mM NaN_3_ in 5 mM HEPES [pH 7.4]) for 1 h at room temperature and 4°C overnight. After fixation, the samples were treated with 2% tannic acid for 1 h, 1% osmium tetroxide for 2 h, 1% thiocarbohydrazide for 30 min, 1% osmium tetroxide overnight, and 2% uranyl acetate for 30 min with washing steps in between. The samples were dehydrated in a graded series of aqueous ethanol solutions (10–100%) and then critical point-dried. Finally, filter were mounted on aluminum stubs, sputtered with gold/palladium and examined in a scanning electron microscope EVO LS10 (Carl Zeiss microscopy GmbH, Oberkochen, Germany).

### 2.3 Transmission Electron Microscopy

Cells were fixed in 1% glutaraldehyde, 4% paraformaldehyde, 50 mM NaN_3_ in 5 mM HEPES for 1 h at room temperature and then at 4°C overnight. Subsequent to embedding the cells in low gelling agarose, cells were postfixed in 2% osmium tetroxide for 2 h at 4°C. After dehydration in graded series of ethanol (20–100%) for 10 min each step with 0.5% uranyl acetate in 70% ethanol for 30 min (at 4°C) in between, the material was embedded in Epon. Sections were cut on an ultramicrotome (Reichert Ultracut, Leica UK Ltd, Milton Keynes, UK), stained with uranyl acetate and lead citrate and analyzed with a transmission electron microscope LEO 906 (Carl Zeiss microscopy GmbH, Oberkochen, Germany).

### 2.4 2D-Gel Electrophoresis

Cytosolic protein extracts were loaded onto IPG-strips in the pH-range 4–7 (GE Healthcare Bio-Sciences AB, Finland) using 100 µg protein for labeled samples and 500 µg for preparative gels. 2D-PAGE was performed as described by Büttner *et al.* [15]. Autoradiography of radioactively labeled gels was performed as previously described [14]. Preparative gels were stained with Coomassie Brilliant Blue as described by Voigt *et al.* [16]. Proteins were excised from preparative gels, digested and the peptide solution spotted onto MALDI targets using the Ettan Spot Handling Workstation (GE Healthcare, UK). Identification was performed using MALDI-TOF-MS/MS (Proteome Analyzer 5800 MDS Sciex, USA) and an in-house *B. pumilus* Jo2 (DSM 14395) database as described by Wolf *et al.* [17]. Protein quantification was done with the Delta2D proteome software (Decodon, Germany).

### 2.5 Microarray Experiment

Total RNA of *B. pumilus* was prepared by the acid phenol method [18] with the modifications described elsewhere [19]. The isolated RNA was treated with DNase (RNase-free DNase Set, Quiagen, Germany) and subsequently concentrated and cleaned (RNA cleanup and concentration Kit, Norgen Biotek, Canada). Quantity of RNA was determined on a microscale spectrophotometer (Nanodrop ND-1000, Peqlab Biotechnologie GmbH, Germany) and RNA integrity was analyzed using a capillary electrophoresis system (Bioanalyzer 2100, Agilent Technologies, USA). Synthesis and purification of fluorescently labeled cDNA was carried out according to Schroeter *et al.* [20] with minor modifications described below. After the labeling and clean-up step [20], 600 ng of respective Cy3- and Cy5 -labeled cDNA were admixed (ad. 44 µl), denaturated and mixed with 11 µl pre-warmed blocking agent and 60 µl hybridization buffer (both Gene expression hybridization kit, Agilent Technologies, USA). 100 µl of the emerging cDNA mixture, respectively, were used for any hybridization. Custom-made *B. pumilus* Jo2 4×44 K gene expression microarrays were obtained from Agilent Technologies (https://earray.chem.agilent.com/earray/), containing 60-mer Oligonucleotide probes (SurePrint technology, Agilent Technologies). Probe design was performed on the chromosome sequence of *B. pumilus* Jo2 (Sequence Intellectual Property of Henkel KGaA). In addition to the annotated open reading frames (ORFs), ORFs were predicted using (i) Glimmer 3.0 [21], (ii) ZCURVE [22], (iii) Genemark HMM [23], and (iv) Prodigal [24]. Predicted ORFs were added to the design provided that: (i) they were non-overlapping with existing ORFs; or (ii) they were in the reverse complementary strand of existing ORFs. On the annotated and predicted ORFs, up to 5 probes were designed. Altogether, a total of 41377 probes were designed by means of OligoWiz 2.1.3 [25] using default parameters for prokaryotic long-mers. The arrays were hybridized and washed according to the manufacturer’s instructions (Two-Color Microarray-Based Gene Expression Analysis Protocol, Agilent Technologies, USA), followed by a last wash step with acetonitrile (Carl Roth GmbH+Co. KG, Germany) for 30 sec. Microarrays were scanned using the Agilent scanner Type G2565CA with high resolution upgrade G2539A and the software Scan Control 8.4.1 (Agilent Technologies, USA). Data were extracted from scanned images using Agilent's Feature Extraction Software (version 10.5.1.1; Agilent Technologies, USA) using default settings. A common reference type of design was employed, and data from three biological replicate hybridizations for each point in time were used for data analysis. Spot signals were normalized using Lowess as described earlier [26]. Next, for each ORF a signal was determined by taking the median signal of the up to 5 probes per ORF. Differential regulation was determined from the biological triplicate measurements by false-discovery rate (FDR) from the Cyber-T p-values [27] by means of multiple testing correction [26]. Differential regulation was defined as a 2-fold or higher differential expression with a FDR cut-off value of 0.05 or lower.

### 2.6 Metabolomic Analysis of Thiols as their Monobromobimane-derivatives

Cells were grown in minimal medium as described above and exponentially grown cells from 10 ml culture medium were harvested before oxidative stress, 10, 30 and 60 min after addition of hydrogen peroxide. The isolation of LMW-thiols for HPLC analysis was performed as described previously [28]. In brief, after centrifugation the cells were washed with 50 mM Tris–HCl (pH 8.0) and resuspended in 50% acetonitrile containing 20 mM Tris–HCl (pH 8.0), 1 mM penicillamine as internal standard and 2 mM monobromobimane (mBBr). Control samples were resuspended without penicillamine and 5 mM N-ethylmaleimide (NEM) was used prior to addition of mBBr. Thiols were extracted at 60°C and directly labeled with mBBr. Labeling reaction was stopped with aqueous methane sulfonic acid in a final concentration of 5 mM. BSmB (monobromobimane-derivative of BSH) standards were synthesized as described previously [7], [29]. For detection and quantification of LMW-thiols, ion pairing HPLC was performed as described before [30]. For absolute quantification the ratio peak area thiol/peak area internal standard was used and an eight-point calibration between 10 nM and 2000 nM was generated.

### 2.7 Prediction of the PerR Consensus Sequence

Prediction of the PerR consensus sequence was done with the PRODORIC® database (http://prodoric.tu-bs.de/vfp/index2.php) release 8.9 [31] using the consensus sequence as described by Fuangthong *et al.* [32].

## Results and Discussion

### 3.1 Effects of H_2_O_2_ on Growth and Cell Morphology

**Figure 1:**
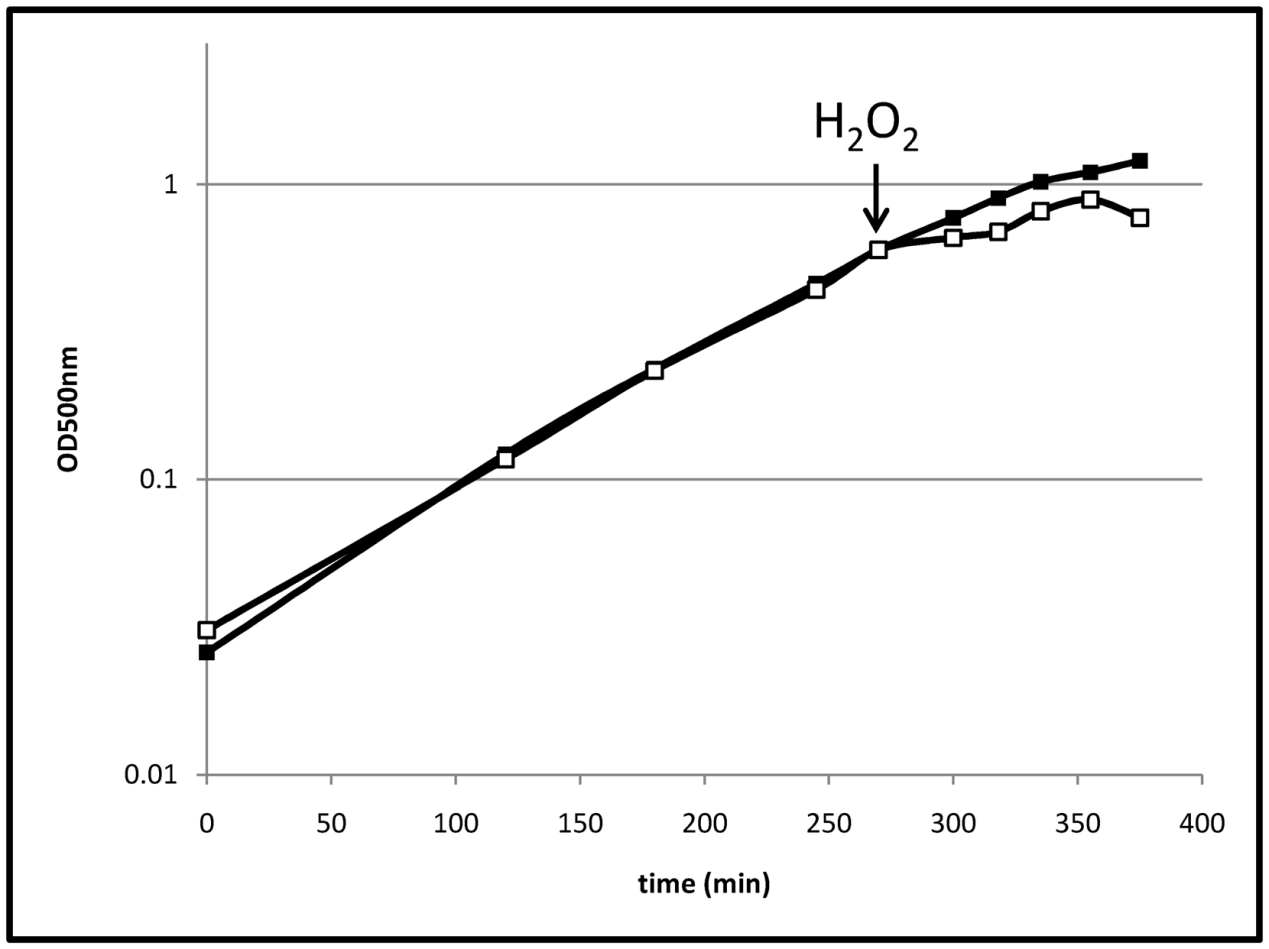
Growth of *B. pumilus.* Growth of *B. pumilus* under control conditions (filled squares) and stressed with 2 mM H_2_O_2_ at OD_500 nm_ 0.6 (empty squares).

**Figure 2:**
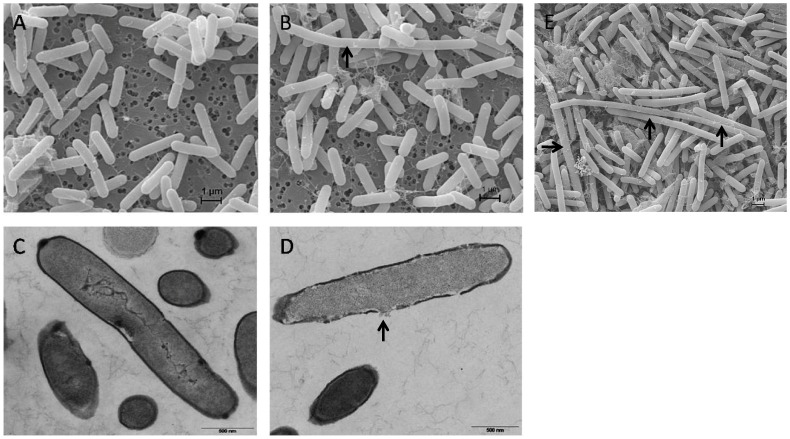
Electron microscopy micrographs. Scanning (A,B,E) and transmission (C,D) electron microscopy micrographs of *B. pumilu*s cells under control conditions (A,C), 30 min (B,D) and 120 min after treatment with 2 mM H_2_O_2_ (E).

Exponentially growing *B. pumilus* cells were treated with 2 mM hydrogen peroxide. Thus, the concentration of H_2_O_2_ that was used to trigger the stress in this study was about 40-fold higher than those used for comparable analyses with *B. subtilis* or *B. licheniformis* [13], [20]. The highest peroxide concentrations allowing growth for *B. subtilis* and *B. licheniformis* were 4 and 1 mM, respectively (Table S1). *B. pumilus* is still able to grow with 20 mM hydrogen peroxide. This indicates a striking resistance of *B. pumilus* against peroxide stress. Compared to unstressed cells, growth was significantly impaired for a short time (approximately 15 min) after the H_2_O_2_ treatment (Figure 1). However, after that time, cells continued to grow for about one hour. An electron microscopy analysis indicated that after exposure to H_2_O_2_ most of the cells are morphologically intact, but some of the cells exhibited major damage of their envelope (Figure 2D). Furthermore, scanning electron microscopy revealed some atypically long cells (up to approximately 10–20% two hours after H_2_O_2_ treatment, Figure 2B, 2E) indicating an impact of hydrogen peroxide on processes involved in cell division.

### 3.2 Global Expression Profile

**Figure 3:**
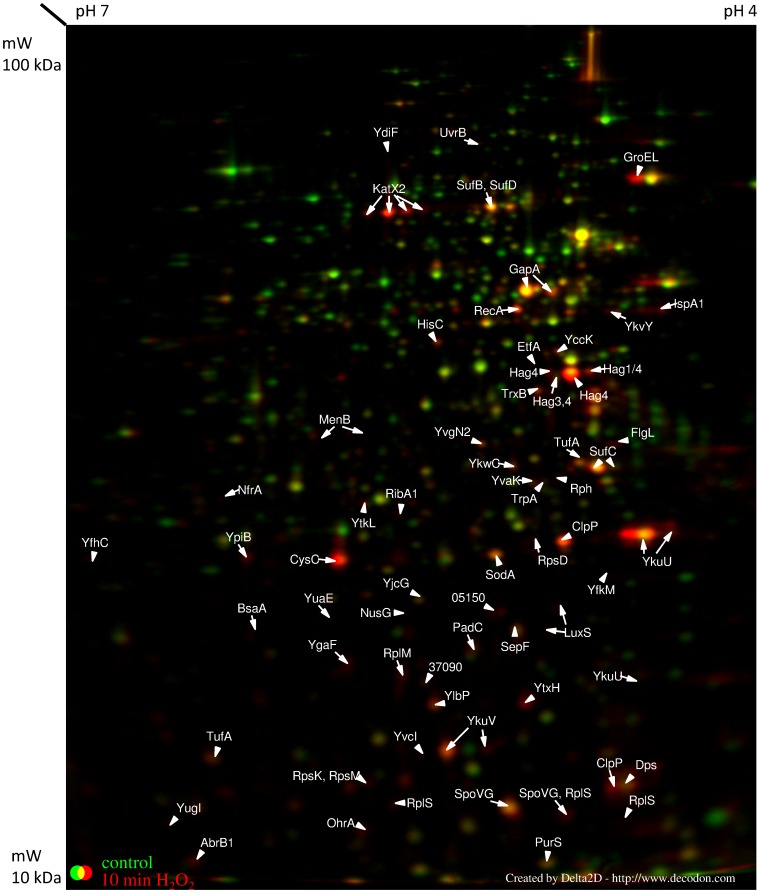
Cytosolic proteome 10 min after H_2_O_2_ treatment. The cytosolic proteome of *B. pumilus* cells 10 min after H_2_O_2_ treatment. Cell samples were labeled with L–[^35^S]-methionine during the exponential growth phase (OD_500 nm_ 0.6), and 10 min after H_2_O_2_ addition. Proteins were separated in a pH gradient 4 (right) –7 (left).

**Figure 4:**
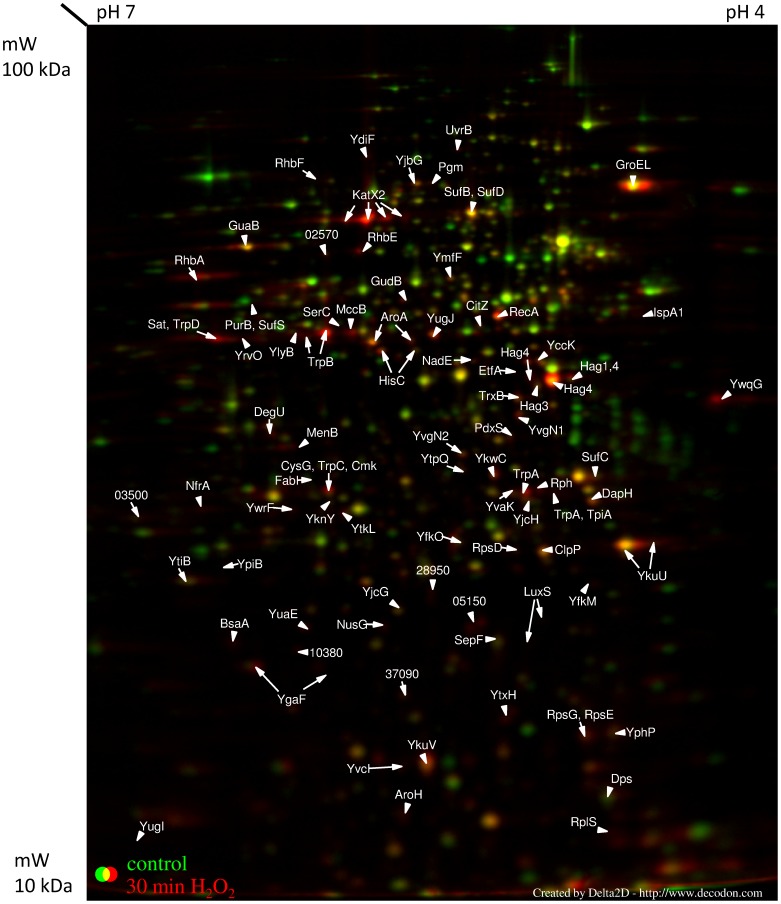
Cytosolic proteome 30 min after H_2_O_2_ treatment. The cytosolic proteome of *B. pumilus* cells 30 min after H_2_O_2_ treatment. Cell samples were labeled with L–[^35^S]-methionine during the exponential growth phase (OD_500 nm_ 0.6), and 30 min after H_2_O_2_ addition. Proteins were separated in a pH gradient 4 (right) –7 (left).

All values presented for up- and downregulation of genes or proteins are fold change values. The analysis of the soluble intracellular proteome of *B. pumilus* revealed 54 significantly upregulated and 111 downregulated proteins 10 min after H_2_O_2_ treatment (with a threshold of 2-fold, Table 1, Table S2, Figure 3). For the visualization of the fast and early response on proteome level, a labeling with ^35^S-methionine was necessary. 30 minutes after initiating the stress, 73 proteins were up- and 59 proteins downregulated (Table 1, Table S2 and S3, Figure 4). Transcriptome analysis revealed an at least 2-fold increased transcription of 181 genes three minutes after treatment with H_2_O_2_; 76 of them were more than 3-fold upregulated. Eight minutes after treatment, the transcription of 558 genes appeared at least 2-fold increased (307 genes with an at least 3-fold increased transcription). Three minutes after the stress, 266 genes were transcribed with an at least 3-fold lower rate than under control conditions, for 296 genes this decreased transcription rate was shown eight minutes after treatment. To indicate quality of the transcriptome results, raw data for individual probes for selected genes (which were not found to be induced in the proteome analysis) are presented in Table S4. These data show similar basal values and changes following addition of hydrogen peroxide for all five probes corresponding to a gene.

**Table 1:** Selected induced genes and proteins.

ORF ID	gene		transcriptome		proteome		Regulon in other *Bacilli*
			3 min	8 min	10 min	30 min	
BPJ13600	zinc-transporting ATPase ZosA	*zosA*	12.74	28.72			perR
BPJ25410	glutamyl-tRNA reductase HemA	*hemA*	3.44	3.99			perR
BPJ25390	porphobilinogen deaminase HemC	*hemC*	2.68	3.90			perR
BPJ25370	delta-aminolevulinic acid dehydratase HemB	*hemB*	2.52	3.72			perR
BPJ25400	putative cytochrome C biogenesis protein HemX	*hemX*	2.86	4.25			perR
BPJ25380	uroporphyrinogen III synthase HemD	*hemD2*	2.68	4.23			perR
BPJ25360	glutamate-1-semialdehyde 2,1-aminomutase HemL	*hemL*	2.75	3.56			perR
BPJ21690	Fur family ferric uptake regulation protein Fur	*fur*	1.92	3.62			perR
BPJ11620	transcriptional regulator Spx	*spxA*	4.14	3.31			perR/spx/sigB
BPJ11610	putative N-acetyltransferase YjbC	*yjbC*	2.41	4.41			perR/spx/sigB/sigM/sigW/sigX
BPJ09760	catalase KatX2	*katX2*	6.96	10.69	15.18	21.09	sigB/sigF
BPJ34450	putative ABC transporter permease YwjA	*ywjA*	1.57	4.47			fur
BPJ30810	hydroxamate siderophore ABC transporter ATP-bindingprotein FhuC	*fhuC1*	1.51	2.46			fur
BPJ30830	hydroxamate siderophore ABC transporter permeaseFhuB	*fhuB1*	1.52	4.01			fur
BPJ30820	hydroxamate siderophore ABC transporter permeaseFhuG	*fhuG1*	1.53	3.20			fur
BPJ08440	ABC transport system permease	*bpj08440*	4.11	7.49			fur
BPJ08430	putative iron complex transport system substratebinding protein	*bpj08430*	4.54	7.43			fur
BPJ08420	putative HTH-type transcriptional regulator	*bpj08420*	3.58	5.59			fur
BPJ08580	putative nitroreductase YfhC	*yfhC*		2.67	5.00	1.10	fur
BPJ08410	ferredoxin–NADP reductase 2	*bpj08410*	3.90	3.83			fur
BPJ37570	AraC family transcriptional regulator/putativeFeuA-like substrate-binding domain ybbB	*ybbB*	4.93	12.84			fur
BPJ37580	iron complex ABC transporter substrate-binding proteinFeuA	*feuA*	3.21	10.04			fur, btr, citB
BPJ37590	putative bacillibactin esterase YbbA	*ybbA*	5.24	18.42			fur/btr/citB
BPJ07970	C56 family peptidase YfkM	*yfkM*	2.94	7.61	7.39	3.09	fur/sigB
RBPU30260	FeS cluster assembly protein SufB	*sufB*	1.87	2.10	1.73		Fe/S cluster biogenesis
RBPU30280	cysteine desulfurase SufS	*sufS*			−1.77	2.69	Fe/S cluster biogenesis
RBPU30290	FeS cluster assembly permease SufD	*sufD*			1.73		Fe/S cluster biogenesis
RBPU30300	FeS cluster assembly ATPase SufC	*sufC*			2.52	2.13	Fe/S cluster biogenesis
BPJ11040	diaminobutyrate–2-oxoglutarate aminotransferase RhbA	*rhbA*			−1.11	11.10	siderophore synthesis
BPJ11080	rhizobactin siderophore biosynthesis protein RhbE	*rhbE*			−1.18	5.28	siderophore synthesis
BPJ11090	rhizobactin siderophore biosynthesis protein RhbF	*rhbF*			−1.72	3.02	siderophore synthesis
BPJ35800	iron complex ABC transporter ATP-binding protein FhuC	*fhuC2*	3.88	7.85			iron uptake
BPJ35810	iron complex ABC transporter permease FhuB	*fhuB2*	3.32	7.15			iron uptake
BPJ35770	putative iron complex ABC transporter permease FhuG	*fhuG2*	2.31	4.39			iron uptake
BPJ35780	putative iron complex ABC transporter substrate-binding protein FhuD	*fhuD*	2.72	5.57			iron uptake
BPJ35830	putative iron transport-associated protein/putative siderophore	*bpj35830*	3.65	5.84			iron uptake
BPJ35840	putative heme uptake protein IsdC	*bpj35840*	4.91	7.62			iron uptake
BPJ35850	putative iron transport-associated protein	*bpj35850*	3.89	6.47			iron uptake
BPJ28430	DinB-like domain-containing protein YuaE	*yuaE*			2.25	2.87	spx
BPJ31980	thioredoxin-disulfide reductase TrxB	*trxB*		3.97	3.93	3.59	spx
BPJ29110	putative NADH-dependent butanol dehydrogenase YugJ	*yugJ*		2.32	1.08	4.60	spx
BPJ19830	methionine sulfoxide reductase MsrA	*msrA*	1.46	2.24			spx
BPJ19820	peptide-methionine sulfoxide reductase MsrB	*msrB*	1.48	2.27			spx
BPJ25870	thioredoxin TrxA	*trxA*	1.40	2.58			spx/ctsR/sigB
BPJ35200	NADPH-dependent nitro/flavin reductase NfrA	*nfrA*		2.50	2.47	5.21	spx/sigD/spo0A
BPJ24450	cystathionine gamma-lyase MccB	*mccB*			−1.58	7.58	spx/cymR
BPJ17710	putative cell division suppressor protein YneA	*yneA*	2.24	44.25			lexA/SOS
BPJ10180	3′-5′ exoribonuclease YhaM	*yhaM*	0.71	2.81			lexA/SOS
BPJ21860	DNA polymerase 4	*polY1*		10.68			lexA/SOS
BPJ32300	excinuclease ABC subunit B	*uvrB*		7.22	2.52	4.29	lexA/SOS
BPJ32290	excinuclease ABC subunit A	*uvrA*	1.49	6.75			lexA/SOS
BPJ25860	excinuclease ABC subunit UvrC	*uvrC*		3.65			lexA/SOS
BPJ17700	repressor LexA	*lexA*	1.55	5.66			lexA/SOS
BPJ17730	DUF896 family protein YnzC	*ynzC*	0.65	8.85			lexA/SOS
BPJ12460	phage-like PBSX protein XkdA	*xkdA*	3.10	17.84			lexA/SOS
BPJ17720	resolvase-like protein YneB	*yneB*	1.38	17.03			lexA/SOS
BPJ10160	putative exonuclease YhaO	*yhaO*		8.76			lexA/SOS
BPJ16880	recombinase RecA	*recA*	1.63	7.22	4.94	9.58	lexA/SOS/comK
BPJ35170	minor extracellular serine protease Vpr	*vpr*	1.58	2.23			lexA/SOS/phoP
BPJ21470	hypothetical protein YpuD	*ypuD*	1.93	7.12			lexA/SOS/sigB/sigM
BPJ10170	putative ATPase YhaN	*yhaN*		8.73			lexA/SOS
BPJ13450	ATP-dependent Clp protease ATP-binding subunit ClpE	*clpE*	2.78	45.41			ctsR
BPJ25460	ATP-dependent protease ATP-binding subunit ClpX	*clpX*		2.67			ctsR
BPJ00800	DNA repair protein RadA	*radA*		10.02			ctsR/sigB
BPJ00760	transcriptional regulator CtsR	*ctsR*		9.40			ctsR/sigB
BPJ00770	transcriptional regulator McsA	*mcsA*		10.26			ctsR/sigB
BPJ31850	ATP-dependent Clp protease proteolytic subunit ClpP	*clpP*	1.79	4.26	8.74	1.73	ctsR/sigB
BPJ00780	putative ATP:guanido phosphotransferase McsB	*mcsB*	1.43	8.87			ctsR/sigB/sigF
BPJ00790	ATP-dependent Clp protease ClpC	*clpC*		6.44			ctsR/sigB/sigF
BPJ00810	DNA integrity scanning protein DisA	*disA*		5.15			ctsR/sigB/sigM
BPJ15470	adenylyl-sulfate kinase CysC	*cysC*			23.93	1.60	cymR
BPJ15480	uroporphyrin-3 C-methyltransferase CysG	*cysG*			1.49	10.53	cymR
BPJ15460	sulfate adenylyltransferase Sat	*sat*			1.37	13.07	cymR
BPJ20800	tryptophan synthase alpha subunit TrpA	*trpA*			2.16	13.51	TRAP
BPJ20810	tryptophan synthase beta subunit TrpB	*trpB*			1.13	13.99	TRAP
BPJ20820	N-(5′-phosphoribosyl)anthranilate isomerase TrpF	*trpF*	1.59	2.09			TRAP
BPJ20830	indole-3-glycerol-phosphate synthase TrpC	*trpC*		2.36	1.49	10.53	TRAP
BPJ20840	anthranilate phosphoribosyltransferase TrpD	*trpD*	1.44	2.72	1.37	13.07	TRAP
BPJ20850	anthranilate synthase component 1	*trpE*		2.61			TRAP
BPJ12980	transcriptional regulator OhrR	*ohrR*		2.54			ohrR
BPJ12970	peroxiredoxin OhrA	*ohrA*	10.66	9.88	6.29	1.30	ohrR
BPJ12990	peroxiredoxin OhrB	*ohrB*		2.10			sigB/ohrR
BPJ19510	putative bacillithiol biosynthesis deacetylase YojG	*yojG*		3.31			bacillithiol-related
BPJ20020	DUF1094 family protein YphP	*yphP*			1.78	2.22	bacillithiol-related
BPJ21140	putative thioredoxin reductase YpdA	*ypdA*	0.67	2.58			bacillithiol-related
BPJ22220	DUF1094 family protein YqiW	*yqiW*		2.58			bacillithiol-related
BPJ31300	glycine betaine/carnitine/choline ABC transporter permease OpuCD	*opuCD*		3.03			glycine betaine transport
BPJ31310	glycine betaine/carnitine/choline ABC transporter substrate-binding protein OpuCC	*opuCC*	0.88	2.62			glycine betaine transport
BPJ31320	glycine betaine/carnitine/choline ABC transporter permease OpuCB	*opuCB*	0.92	2.74			glycine betaine transport
BPJ31330	glycine betaine/carnitine/choline ABC transporter ATP-binding protein OpuCA	*opuCA*	0.94	2.46			glycine betaine transport
BPJ02950	glycine betaine ABC transporter ATP-binding protein OpuAA	*opuAA*	2.76	10.75			glycine betaine transport
BPJ02960	glycine betaine ABC transporter membrane protein	*opuAB*	2.41	10.19			glycine betaine transport
BPJ02970	glycine betaine ABC transporter substrate-binding protein	*opuAC*	2.37	7.48			glycine betaine transport
BPJ29360	Na+/H+ antiporter subunit MrpA	*mrpA*		4.57			sodium transport
BPJ29370	Na+/H+ antiporter subunit MrpB	*mrpB*		4.77			sodium transport
BPJ29380	Na+/H+ antiporter subunit MrpC	*mrpC*		3.60			sodium transport
BPJ29390	Na+/H+ antiporter subunit MrpD	*mrpD*	1.48	3.87			sodium transport
BPJ29400	Na+/H+ antiporter subunit MrpE	*mrpE*	1.51	3.20			sodium transport
BPJ29410	Na+/H+ antiporter subunit MrpF	*mrpF*	1.72	3.38			sodium transport
BPJ29420	Na+/H+ antiporter subunit MrpG	*mrpG*	1.96	2.60			sodium transport

Selected genes and proteins that are induced in H_2_O_2_ treated *B. pumilus* cells.

Genes and proteins are listed, which could be assigned to putative regulons known from other *Bacilli*. Complete lists of upregulated as well as downregulated genes/proteins is given in supporting information Tables S2 and S3. For transcriptome, selected genes are shown for 3 and 8 minutes after stress compared to the control conditions (0 min). For a complete list of induced and repressed genes see Table S3. Differential regulation was determined from the biological triplicate measurements by false-discovery rate (FDR) from the Cyber-T p-values [27] by means of multiple testing correction [26]. Differential regulation was defined as a two-fold or higher differential expression with a FDR cut-off value of 0.05 or lower. Protein quantification was performed by the Delta 2D software (Decodon) from 3 biological replicates with a FDR cut-off value of 0.05 or lower.

To compare the physiological changes in H_2_O_2_ treated *B. pumilus* cells with the oxidative stress responses of other organisms, the upregulated genes and proteins were assigned to putative regulons known from related organisms like *B. subtilis* and *B. licheniformis* [13], [20]. 139 of the upregulated genes and proteins could be assigned to these putative regulons (Table S2). The thus classified genes and proteins identified in this study are summarized and discussed below.

### 3.3 PerR Regulon

The PerR regulon is known to be highly induced by oxidative stress caused by hydrogen peroxide and paraquat [13]. As shown previously for *B. licheniformis*, the *B. pumilus* genome encodes a PerR regulator protein with a high level of identity (93%) to the PerR-protein known from *B. subtilis* [20]. Transcription of the *perR* gene was significantly increased immediately after stress (Table 1). This indicates a regulation mechanism of PerR in H_2_O_2_ treated *B. pumilus* cells that is similar to the de-repression model reported for *B. subtilis* [33].

In our study genes assigned to a putative PerR regulon, including those encoding the regulator proteins Fur and SpxA as well as the zinc-uptake protein ZosA, the heme biosynthesis complex HemABCD2LX and the general stress protein YjbC were significantly induced at transcriptional level (Table 1).

Strikingly, some of the PerR-regulated genes exhibiting the highest induction in *B. subtilis* cells subjected to hydrogen peroxide, are absent from the genome of the *B. pumilus* strain used in our study, as well as from a previously published *B. pumilus* genome [34]. This applies e.g. for the genes encoding the catalase KatA and the DNA-protection protein MrgA. Furthermore, *B. pumilus* lacks not only the genes *ahpC* and *ahpF*, encoding subunits of the alkyl hydroperoxide reductase, but there are no genes annotated with this function in the genome.

Instead of KatA, a gene annotated as catalase KatX2 (53% sequence similarity to *B. subtilis* KatX) was significantly induced in *B. pumilus* cells at transcriptional and translational level (up to 10 and 20-fold, respectively, Table 1). Thereby, KatX2 was one of the proteins with the highest induction rates detected. *B. subtilis* and *B. licheniformis* subjected to hydrogen peroxide exhibit a more than 100-fold induction of KatA [13], [20]. KatX2 comprises about 0.38% of the cytoplasmic protein present in the gel before addition of hydrogen peroxide. The values for *B. subtilis* and *B. licheniformis* are 0.13% in both strains (personal communication C. Scharf, B. Voigt). After addition of hydrogen peroxide KatX2 comprises about 3.8% of the cytoplasmic protein. This is comparable to the value of 3.6% for *B. licheniformis* (personal communication B. Voigt) but higher than the value for *B. subtilis* (1.2%, personal communication C. Scharf). These values indicate that in *B. pumilus* there is a higher synthesis of KatX2 already in unstressed cells compared to *B. subtilis* and *B. licheniformis* KatA explaining the lower induction rate. In *B. subtilis*, KatX is the major spore catalase and under control of SigB and SigF [35], [36]. We detected a *B. subtilis* PerR consensus sequence [32] containing 2 mismatches about 90 bases in front of the start codon of KatX2 indicating a possible involvement of PerR in its regulation.

### 3.4 Fur Regulon and Fe-metabolism

The PerR-regulated *fur* gene *of B. pumilus*, shows 95% similarity to the *fur* gene known from *B. subtilis* and was induced 3.6-fold after stress [32]. The regulator protein Fur of *B. subtilis* controls the expression of genes responsible for iron uptake [37]. Immediately after exposure to H_2_O_2_, cytosolic iron concentration is considerably reduced to prevent the formation of OH^•^ by the Fenton reaction [13]. Upregulation of the Fur-controlled genes may be a reaction of the cells to optimize iron uptake in order to face the resulting iron limitation. Alternatively it might be that Fur is H_2_O_2_ sensitive as it is in *E. coli* [38].

Nine genes of a putative Fur regulon showed a significantly increased expression in *B. pumilus* cells after H_2_O_2_ treatment, including the ABC transporter system *fhuB1C1G1* (Table 1). The *fhuC* gene was induced by H_2_O_2_ in *B. subtilis* and *B. licheniformis*, too [13], [20]. Further Fur regulon member genes known to be induced by H_2_O_2_ in *B. subtilis* showing an induction in our study were *ykuN*, *ykuP* (flavodoxins) and the hypothetical protein *ykuO*. With an about 30-fold higher mRNA level 8 minutes after treatment, these were among the highest upregulated genes in this putative regulon. The putative nitroreductase YfhC, also induced in H_2_O_2_ stressed *B. subtilis* cells, was the only member of the putative Fur regulon we observed to be upregulated at translational level.

The gene *ywjA*, encoding another ABC transporter of yet unknown function, the peptidase encoding gene *yfkM* and the bacillibactin esterase encoding gene *ybbA* were upregulated, too. These genes are Fur-regulated in *B. subtilis*, but they were not upregulated by H_2_O_2_ in this organism [13], [39]. In *B. subtilis* and *B. licheniformis*, the siderophore biosynthesis complex encoded by *dhbACEBF* was strongly upregulated by H_2_O_2_. In our study, these genes showed no significant changes in their expression level.

Other genes that exhibited higher transcription rates after H_2_O_2_ treatment were the iron ABC transporter protein encoding gene *feuA* and its upstream-located regulator *ybbB* (renamed *btr* in *B. subtilis*) [40]. Unlike *B. subtilis*, the *B. pumilus* genome encodes a second Fhu-related iron uptake system. Our study showed an induction of the genes encoding FhuC2-FhuB2-BPJ35820 as well as *fhuG2* and *fhuD* immediately after subjecting the cells to the stress. Two further putative iron transporter systems, *bpj35830*-*bpj35840*-*bpj35850* and *bpj08420*-*bpj08430*-*bpj08440*, were induced, too. The proteins encoded by the latter genes showed no significant homology to any protein known from related *Bacillus* species.

Furthermore, the proteomic approach revealed a strong induction of the siderophore synthesis proteins RhbA, RhbE and RhbF, encoded by the *rhbABCDEF*-operon (Table 1). A rather slight induction at the translational level was shown for the iron/sulfur cluster biogenesis proteins SufB, SufS, SufD and SufC as previously shown for *B. licheniformis* [20]. The *sufU* gene was found to be only slightly upregulated at the mRNA level.

### 3.5 Spx Regulon and Bacillithiol

Another regulator protein assigned to the putative PerR regulon is SpxA, controlling the expression of the Spx regulon in *B. subtilis* [41], [42]. This gene exhibited an about 4-fold increased transcription rate in H_2_O_2_ stressed *B. pumilus* cells. Some of the genes and proteins attributed to a putative Spx regulon in *B. pumilus* appeared to have rather moderately increased expression rates or were not induced after H_2_O_2_ treatment.

In our study we detected six genes of a putative Spx regulon to be induced following H_2_O_2_ treatment (Table 1). The proteins encoded by three of them, nitro/flavinreductase NfrA, putative NADPH-dependent butanol dehydrogenase YugJ and thioredoxin-disulfide reductase TrxB, were induced in H_2_O_2_ treated cells, too. Upregulation of *msrAB* (methionine sulfoxide reductase operon) and *trxA* (thioredoxin) was detected at transcriptional level only. The proteins TrxA and TrxB are described to act in direct detoxification of hydrogen peroxide [43]–[45]. Cystathionine gamma-lyase MccB and DinB-like domain-containing protein YuaE showed an induction only at proteome level.

The Spx-regulated *srf* operon, mediating competence and metabolic functions in *B. subtilis*, is absent in the *B. pumilus* genome as shown before for *B. licheniformis* [42], [46], [47].

**Figure 5:**
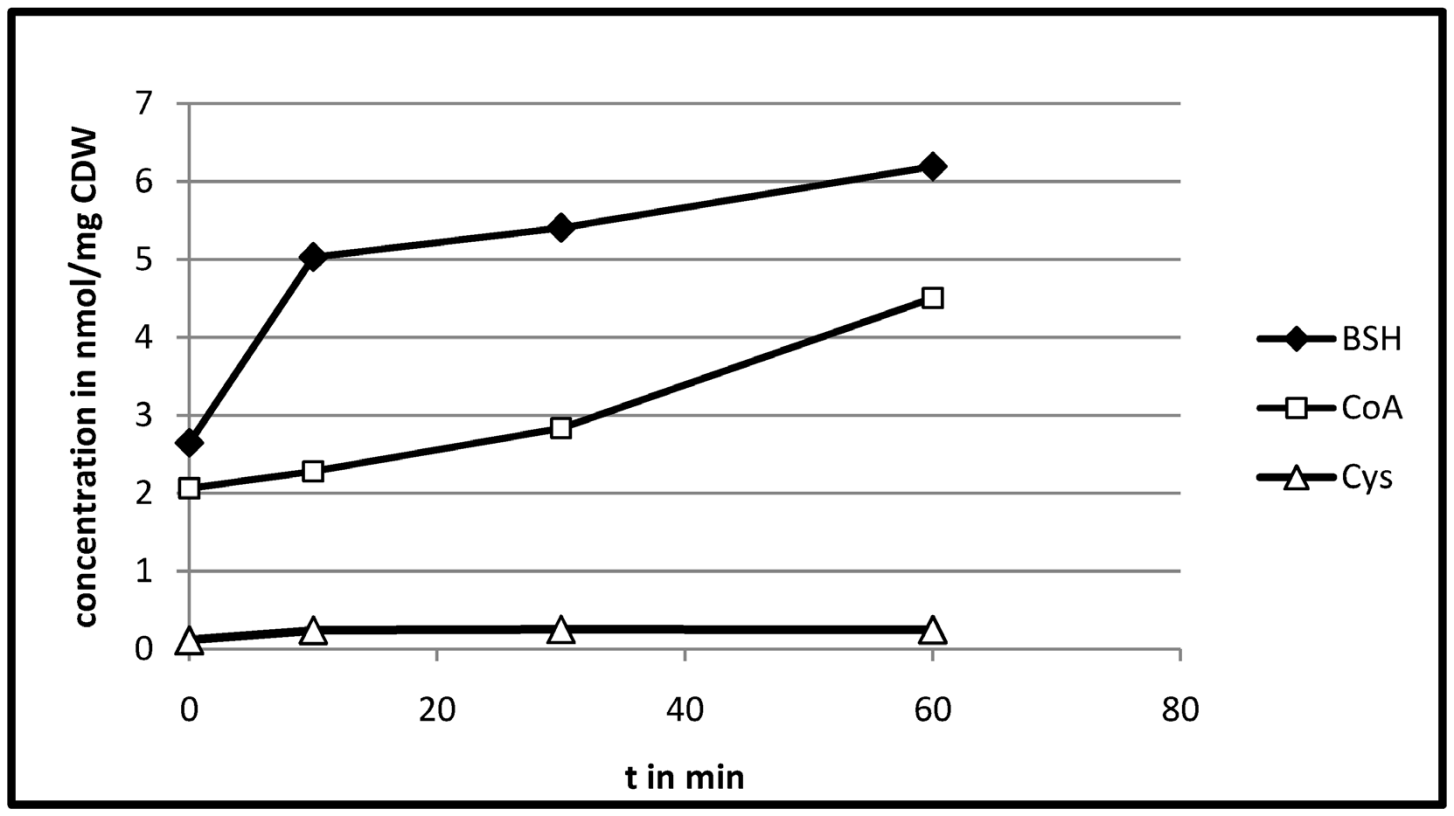
Concentration of thiol compounds in *B. pumilus* cells. Cytosolic concentration of bacillithiol (BSH), CoA and cysteine (Cys) per mg cell dry weight (CDW) during the exponential growth phase (OD_500 nm_ 0.6 at 0 min) and 10, 30 and 60 min after H_2_O_2_ treatment.

We noticed an increased transcription of *ypdA* and *yqiW* as well as an induction of the *yphP* gene product (Table 1). These genes co-occur with bacillithiol (Cys-GlcN-malate, BSH) synthesis genes [48]. However, only one gene encoding a protein involved in bacillithiol synthesis, *yojG* was transcribed at a slightly elevated level (Table S2). Bacillithiol is one of the major thiols in *B. subtilis* and known to be involved in resistance against organic peroxide stress and disulfide stress [7], [49], [50]. For further investigation, we analyzed the cytosolic metabolome of H_2_O_2_ treated *B. pumilus* cells concerning the concentration of thiol compounds. Our analysis revealed a bacillithiol level of 2.6 nmol per mg cell dry weight already under control conditions. Similar BSH concentrations have been detected in *B. subtilis* (0.6–2.2 nmol per mg) [7], [48], [51]. Ten minutes after H_2_O_2_ treatment, the cytosolic concentration of bacillithiol increased to 5 nmol per mg cell dry weight (Figure 5). The increase continued up to a concentration of about 6.2 nmol per mg cell dry weight 60 minutes after stress. Since only one bacillithiol synthesis gene (*yojG*, renamed *bshB2* in *B. subtilis*) was slightly upregulated, increase of bacillithiol concentration in the cells might be regulated allosterically, for example, by an oxidation of the BSH pool leading to a relief of feedback inhibition. [52], [53].

### 3.6 SOS Regulon

H_2_O_2_ treatment leads to the formation of OH^•^ by Fenton reaction, which exhibits a high DNA-damaging potential. Lowering the concentration of iron in the cells reduces this threat. As a result, *B. subtilis* and *B. licheniformis* cells subjected to oxidative stress caused by H_2_O_2_, induced the SOS regulon, regulated by the proteins RecA and LexA, responsible for repair of DNA [13], [20], [54], [55].

The proteomic analysis displayed the induction of two proteins, excinuclease subunit UvrB and the recombinase RecA, assigned to a putative SOS regulon in *B. pumilus* following H_2_O_2_ treatment (Table 1). The transcriptomic approach added further 13 upregulated genes belonging to this putative regulon; among them the excinuclease subunits encoding genes *uvrA* and *uvrC*. The operon *yneABynzC*, induced by H_2_O_2_ and involved in suppression of cell division in *B. subtilis*, was also strongly induced in our study [13], [56]. This might be an explanation for the formation of atypically long cells as described above. Showing an about 44-fold increased transcription rate, *yneA* belongs to the strongest induced genes observed in our study. Furthermore, the putative DNA double-strand break repair cluster *yhaONM* exhibited a significantly higher transcription rate following H_2_O_2_ addition [57].

### 3.7 CtsR Regulon

The CtsR regulon, mediating repair and/or degradation of misfolded and damaged proteins, was induced by several oxidative stressors in *B. subtilis* and *B. licheniformis* [13], [20], [58]. In our study, we detected an upregulation of nine genes assigned to a putative CtsR regulon in *B. pumilus* indicating a significant impact of H_2_O_2_ on protein quality (Table 1). The operon *ctsR*-*mcsAB*-*clpC* was transcribed with significantly higher intensity after the addition of H_2_O_2_ as well as the genes *clpE*, *clpX* and *clpP*, encoding members of the proteolytic complex. Only ClpP was observed to be induced at the protein level. Furthermore, the DNA repair protein encoding gene *radA* and the DNA integrity scanning protein encoding gene *disA* showed higher transcription rates compared to control conditions.

### 3.8 SigB Regulon

Besides the induction of the above described putative regulons more or less directly associated to oxidative stress, H_2_O_2_ treated cells exhibited an upregulation of 47 genes known to be under control of the general stress sigma factor SigB in *B. subtilis* (Table 1) [59], [60]. A part of a putative SigB-regulon in *B. pumilus* detected to be upregulated in our study was the *sigB* gene itself with its signal cascade genes *rsbRSTUVW* and *rsbX* indicating an activation of the putative regulon via the general stress response cascade known from *B. subtilis* [61].

Another of these putative SigB-dependent genes, encoding the putative universal stress protein NhaX, showed the highest induction rate detected in this study (more than 60-fold). Further strongly upregulated genes are the regulator protein encoding gene *mgsR* and *ydaG* (general stress protein), both also detected to be induced in H_2_O_2_ stressed *B. licheniformis* cells [20]. The upregulated genes *mgsR* and *ydaG* encode proteins with still unknown functions. Six of the upregulated putative SigB-dependent genes could be also detected to be induced in the proteomic approach. The putative general stress protein YtxH is among the strongest induced proteins (about 14-fold). The putative iron storage/DNA protecting protein Dps, providing peroxide resistance in *B. anthracis*, was induced in H_2_O_2_ treated *B. pumilus* cells, too [62].

### 3.9 CymR Regulon

The results of our study showed an upregulation of several proteins belonging to a putative CymR regulon. In *B. subtilis*, it is described to be involved in regulation of the sulfur metabolism [63]. An induction of genes belonging to this regulon has been shown in cells afflicted with oxidative stress caused by paraquat, but not stress caused by H_2_O_2_ [13]. Our proteome study showed a strong induction of three putatively CymR-regulated proteins. The adenylyl-sulfate kinase (CysC) was with an induction of about 24-fold the strongest induced protein. An upregulation of the sulfate adenylyltransferase (Sat) catalyzing sulfate assimilation to 3′-phospho-adenylylsulfate was also detected (Table 1). Further proteins involved in cysteine biosynthesis were not significantly upregulated. The third upregulated protein is the uroporphyrin-3 C-methyltransferase (CysG). This enzyme catalyzes a reaction in a branch in the heme pathway producing precorrin2. An induction of the enzymes that continue the pathway from precorrin2 to siroheme could not be detected.

### 3.10 Other *B. pumilus* Upregulated Genes/proteins

The OhrR-regulated peroxiredoxin-encoding gene *ohrA* is reported to be involved in organic peroxide resistance in *B. subtilis* [64]. Following H_2_O_2_ treatment, there was no induction of this gene observed in *B. subtilis* and *B. licheniformis* [13], [20]. In our study, we observed a strongly induced expression of this gene at transcriptional and translational level indicating an involvement of this peroxiredoxin in the H_2_O_2_ resistance of *B. pumilus* (Table 1). Transcription of the other organic peroxide resistance peroxiredoxin (*ohrB*) as well as their regulator gene *ohrR* was also slightly induced in hydrogen peroxide treated *B. pumilus* cells.

H_2_O_2_ treatment induced some additional regulator genes. One of them is *fadR*, encoding a regulator protein mediating fatty acid degradation in *B. subtilis* [65]. Two genes putatively controlled by FadR, *etfAB* - encoding the electron transfer flavoprotein alpha and beta subunit, were also induced (Table S2). Another regulator, AbrB1, controlling the expression of genes induced by transition from exponential to stationary growth in *B. subtilis* [66], was induced at transcriptional and translational level. Similar results, but with significantly higher induction rates in the proteomic approach, were observed for the AbrB1-regulated peroxiredoxin YkuU and thiol-disulfide oxidoreductase YkuV. Furthermore, several putative regulator genes with still unknown targets were observed to be upregulated. *Bpj13620*, *bpj17020* and *ydcI* showed the highest changes in their expression rates. Genes encoding a sensor kinase and a response regulator forming the two-component system YhcYZ were significantly induced directly after H_2_O_2_ treatment. Its function is also unknown.

Several genes and proteins involved in transport processes were detected to be upregulated following H_2_O_2_ stress (Table 1, S2). H_2_O_2_ treatment caused an upregulation of the sodium uptake system *natAB* and the *mrpABCDEFG* cluster. This operon encodes a sodium excretion system that is considered to be the major sodium excretion system in bacteria and acts in pH homeostasis and multiple resistances in *B. subtilis* [67], [68].

Strikingly, transcription of the glycine betaine uptake system consisting of *opuAA*-*AB*-*AC* and *opuCA*-*CB*-*CC*-*CD* was observed to be significantly induced after treatment, indicating that H_2_O_2_ impacts osmotic homeostasis in *B. pumilus* cells [69]. Furthermore, it is worth to mention that H_2_O_2_ induced expression of a putative TRAP regulon in *B. pumilus* cells. An upregulation of the tryptophan-synthesis operon *trpABFCDE* as well as histidinol-phosphate aminotransferase HisC was observed in our analysis. However, neither addition of tryptophan nor addition of glycine betaine before peroxide treatment brought forth better growth or survival of stressed *B. pumilus* cells (data not shown).

### 3.11 Downregulated Genes/proteins

As shown for many other organisms, the adaptation mechanism of *B. pumilus* cells to oxidative stress includes also a downregulation of vegetative cellular functions. Most of the down-regulated genes encode proteins involved in main metabolic pathways. As shown for *B. subtilis* and *B. licheniformis*, expression of the purine and pyrimidine synthesis genes was downregulated as well as genes involved in synthesis of arginine (Table S3) [13], [20]. Contrary to *B. subtilis* and *B. licheniformis*, a repression of histidine synthesis genes was not observed. Instead, isoleucine and leucine synthesis genes were expressed in lower amounts following H_2_O_2_ treatment. This repression might due to the iron sparing response described by Gaballa et al. [70]. Repression of enzymes involved in branched chain amino acid synthesis has been found during iron starvation in *B. subtilis* [37]. Furthermore, we observed a reduced expression of most of the aminoacyl-tRNA-synthetases, with the exception of tryptophanyl-tRNA-synthetase *trpS*, which matched the upregulation of the tryptophan operon.

Strikingly, a stringent response, i.e. a downregulation of ribosomal proteins or elongation factors like *fusA*, *tsf* or *tufA*, as described for other organisms (*B. subtilis*, *B. licheniformis*, *E. coli*) could not be detected in *B. pumilus* [13], [20], [71].

## Conclusion

The combination of proteomics and transcriptomics revealed a specific adaptation of *B. pumilus* cells caused by the oxidative stress trigger H_2_O_2_. Although many of the induced genes and proteins could be assigned to well-known oxidative stress regulons like PerR, CtsR and Fur, there are particular mechanisms detectable which seem to be involved in the remarkable oxidative stress resistance of *B. pumilus*. The concentration of H_2_O_2_ that was used to trigger the stress in our study was about 40-fold higher than those used for comparable analysis of *B. subtilis* or *B. licheniformis*. Our study could enlighten several points at which the peroxide stress response of *B. pumilus* cells is different from its Gram-positive relatives. It is suggested that the catalase KatA is replaced by the catalase KatX2. Furthermore, our study revealed an induction of genes that are highly correlated to bacillithiol synthesis indicating an involvement of bacillithiol in the peroxide stress response of *B. pumilus*. Metabolome analysis demonstrated a basal level of this protective metabolite but also an increase of the cytosolic bacillithiol concentration during peroxide stress. Furthermore, a considerable set of H_2_O_2_ induced unique proteins with so far unknown function could be identified in this study. These proteins are worth to address in follow up studies to elucidate their specific role in the oxidative stress adaptation of this organism. Finally, since *B. pumilus* is an organism of industrial interest, understanding its oxidative stress response and defining marker genes for the analysis of fermentation processes is important to prevent possible negative influences on the process and the product quality.

## Supporting Information

Table S1 **Determination of minimal inhibition concentration of hydrogen peroxide.**
(XLSX)

Table S2 **Genes and proteins that are upregulated after addition of hydrogen peroxide.**
(XLSX)

Table S3 **Genes and proteins that are downregulated after addition of hydrogen peroxide.**
(XLSX)

Table S4 **Individual signal on the array for five probes for selected genes.**
(XLSX)
