# Expression of Concern: *Bacillus pumilus* Reveals a Remarkably High Resistance to Hydrogen Peroxide Provoked Oxidative Stress

**DOI:** 10.1371/journal.pone.0100716

**Published:** 2014-07-10

**Authors:** The PLOS ONE Editors

Following publication of this article, a reader made a request for the *Bacillus pumilus* Jo2 strain employed in this study.

The *PLOS ONE* policy governing the sharing of materials and data that applies to articles submitted before March 3, 2014, requires that authors agree to make freely available any materials and data described in their publication that may be reasonably requested for the purpose of academic, non-commercial research.

The journal evaluated the request and concluded that it falls within the requirements of the policy above. The journal has contacted the authors, who indicated that Henkel, the company that provided the strain, will not share the strain or its genome sequence with other researchers and that they were not aware of this restriction on the availability of the strain at the time at which the study was conducted. This is not in line with the journal's policy and the authors' declaration of adherence to its requirements.

The editors are issuing this Expression of Concern to alert readers about the fact that the *Bacillus pumilus* Jo2 strain employed in this study is not available, and thus that the article is in breach of the journal's editorial policy.

## References

[1] Handtke S, Schroeter R, Jürgen B, Methling K, Schlüter R, et al (2014) *Bacillus pumilus* Reveals a Remarkably High Resistance to Hydrogen Peroxide Provoked Oxidative Stress. PLoS ONE 9(1): e85625 doi:10.1371/journal.pone.0085625 24465625 10.1371/journal.pone.0085625 PMC3896406

